# Human brucellosis: recent advances and future challenges

**DOI:** 10.1186/s40249-020-00715-1

**Published:** 2020-07-23

**Authors:** David O’Callaghan

**Affiliations:** 1 Bacterial Virulence and Infectious Disease, University of Montpellier, INSERM, Nimes, France; 2 Brucellosis National Reference Centre (CNR), Microbiology Laboratory, Caremeau University Hospital, Nimes, France

Bacteria of the genus *Brucella* cause brucellosis, one of the world’s neglected zoonotic diseases. It is a disease of poverty; infections of livestock have a huge socioeconomic cost while human brucellosis starts as a debilitating acute infection that can be come chronic with many complications. The figure of 500 000 new human cases each year is regularly cited in reviews and research papers, however this is a vast under estimation; many of the most affected countries do not have the infrastructure for diagnosis and the broad spectrum of symptoms are shared with other febrile infections.

Considering the importance of the disease, the international brucellosis research community is small; we cover a wide range of research interests ranging from veterinary and human medicine to molecular genetics, cell biology and immunology. Much work is centred on the deciphering how *Brucella* cause disease in animals and man; how they enter and survive in host cells, how they use their VirB Type IV secretion system to deliver effectors that modulate host cell biology and immune response.. This will lead to the identification of new strategies for vaccine development, targets for diagnostic and prognostic tests and the development of new therapies. However, as noted in the first few lines of this editorial, brucellosis is a disease of poverty; and it is important that, especially for disease surveillance, diagnosis, control and treatment, that we work for solutions that are relevant and applicable to the countries where the disease is really a problem.

Here I will list a few points that highlight a small selection of the recent practical advances and some of the major challenges.

## Brucellosis control

In endemic areas, control of brucellosis is the first challenge. The only way to control human brucellosis is to control the animal disease and stop passage to man. Brucellosis has been controlled or even eradicated in a small number of wealthy countries, by long and costly programs of animal vaccination followed culling of infected animals at later stages. Food hygiene, especially pasteurization of milk is of great importance to prevent human infections. Excellent reviews by JM Blasco [1, 2] discus this in detail.

Control of a disease such as brucellosis requires a ‘One Health’ approach. Animal and human health must work together with the livestock holders and programs established inform and educate the population at risk. Strong implication of political decision makers is essential. If not yet established, surveillance of human and animal populations should be implemented.

Vaccination programs need good vaccines. Two live vaccines, *B. melitensis* Rev. 1 and *B. abortus* S19 have been used over past decades with great success for, respectively, small ruminant and bovine brucellosis control programs throughout the world. *B. abortus* RB51 is also proposed as a vaccine for bovine brucellosis to be used in the final stages of control programs in conjunction with test and slaughter. None of the available vaccines are perfect; they cause abortion in target and non-target animals, can be shed by immunized animals and all can cause brucellosis in humans. RB51 is also resistant to rifampicin, one of the drugs of choice to treat human brucellosis. We need new effective vaccines that are safe for both animals and humans. There are many projects aiming to improve the efficiency and safety of existing vaccines and to develop new vaccines. There is currently an international call for development of a new brucellosis vaccine with a substantial prize for the first new vaccine licensed (https://brucellosisvaccine.org/). Here, the focus is on a vaccine that will be beneficial in endemic regions. In past years, work has been strongly oriented to vaccines, and diagnostic tools to solve problems in countries with low levels of incidence (generally rich countries in the later stages of control programs). This has led to the DIVA concept (distinguishing between infected and vaccinated animals). While not relevant in a country with high prevalence and no infrastructure to test animals [2], DIVA compliance would make a vaccine more attractive in rich countries and therefore more commercially viable for the manufacturer. The general methods to create a DIVA vaccine have been to remove an immunogenic antigen from the vaccine (such as the loss of O-antigen in RB51). This may reduce efficiency of the vaccine (hence the attempts to restore O-antigen production to RB51 [3]) and mean that accidental human infections are not detected [4]. A more efficient method could be to express an unrelated immunogenic protein in the vaccine strain that would induce a detectable serological response not seen in infected animals. This approach has used for viral vaccines for some time [5] and has recently been used with S19 by the Moreno group in Costa Rica [6].

## Strain identification and molecular epidemiology

MALDI-TOF mass spectrometry is revolutionizing the clinical diagnostic laboratory, but not all machines can identify *Brucella*. We developed a spectral database allowing *Brucella* to be identified by the bioMérieux VITEK system [7] and a safe, rapid protocol for solvent inactivation before analysis. Solvent inactivated bacteria are stable for several days, allowing transport to a centre with a machine [8].

Multilocus sequence typing (MLST) and Multiple-Locus Variable number tandem repeat Analysis (MLVA) is now used throughout the world for molecular epidemiology. These studies are showing how *B. melitensis* and *B. abortus* strains have been transported across the world by animal trade and will be important in the future control programs [9, 10]. The advances in sequencing technology will lead to the generalisation and automatization of in silico core genome MLST and MLVA analysis using whole genome sequences.

### Scientific Integrity

The final challenge is not scientific. An increasing number of cases where scientific integrity was not respected. While some may be unintentional, others include data falsification, image manipulation and plagiarism. Scientific advancement is built on the foundations of solid published data. ‘Fake Science’ is as dangerous as ‘Fake News’. It is our responsibility to ensure that all published papers in the field are honest.

I hope that this thematic series of *Infectious Diseases of Poverty*: Control strategy and case management of human brucellosis, will be the opportunity to report scientific advances in our understanding of brucellosis and describe how they are helping us control this disease.

## Data Availability

Not applicable.

## Abbreviations

DIVA: Differentiation of infected from vaccinated animals
MLST: Multilocus sequence typing
MLVA: Multiple-Locus Variable number tandem repeat Analysis
MALDI-TOF: Matrix Assisted Laser Desorption Ionization - Time of Flight
